# A Simulation of the Effect of External and Internal Parameters on the Synthesis of a Carbyne with More than 6000 Atoms for Emerging Continuously Tunable Energy Barriers in CNT-Based Transistors

**DOI:** 10.3390/nano13061048

**Published:** 2023-03-14

**Authors:** Chi Ho Wong, Yan Ming Yeung, Xin Zhao, Wing Cheung Law, Chak Yin Tang, Chee Leung Mak, Chi Wah Leung, Lei Shi, Rolf Lortz

**Affiliations:** 1 Department of Industrial and Systems Engineering, The Hong Kong Polytechnic University, Hong Kong 999077, China; 2 Research Institute for Advanced Manufacturing, The Hong Kong Polytechnic University, Hong Kong 999077, China; 3 School of Science, The Hong Kong University of Science and Technology, Hong Kong 999077, China; 4 Department of Biomedical Engineering, The Hong Kong Polytechnic University, Hong Kong 999077, China; 5 Department of Applied Physics, The Hong Kong Polytechnic University, Hong Kong 999077, China; 6 State Key Laboratory of Optoelectronic Materials and Technologies, Nanotechnology Research Center, School of Materials Science and Engineering, Sun Yat-sen University, Guangzhou 510275, China; 7 Department of Physics, The Hong Kong University of Science and Technology, Hong Kong 999077, China

**Keywords:** carbyne, carbon nanotube, Monte Carlo simulations

## Abstract

Transistors made up of carbon nanotube CNT have demonstrated excellent current–voltage characteristics which outperform some high-grade silicon-based transistors. A continuously tunable energy barrier across semiconductor interfaces is desired to make the CNT-based transistors more robust. Despite that the direct band gap of the carbyne inside a CNT can be widely tuned by strain, the size of the carbyne cannot be controlled easily. The production of a monoatomic chain with more than 6000 carbon atoms is an enormous technological challenge. To predict the optimal chain length of a carbyne in different molecular environments, we have developed a Monte Carlo model in which a finite-length carbyne with a size of 4000–15,000 atoms is encapsulated by a CNT at finite temperatures. Our simulation shows that the stability of the carbyne@nanotube is strongly influenced by the nature and porosity of the CNT, the external pressure, the temperature, and the chain length. We have observed an initiation of the chain-breaking process in a compressed carbyne@nanotube. Our work provides much-needed input for optimizing the carbyne length to produce carbon chains much longer than 6000 atoms at ~300 K. Design rules are proposed for synthesizing ~1% strained carbyne@(6,5)CNT as a component in CNT-based transistors to tune the energy barriers continuously.

## 1. Introduction

Continuously adjusting the band gap of semiconductors is expected to revolutionize semiconductor technology [1,2]. The charge current flowing through semiconductor devices such as p-n junctions, field-effect transistors, and bipolar junction transistors are related to the energy barrier across the transition region between semiconductor interfaces [2]. Recently, carbon nanotube (CNT) transistors outperform some high-class silicon-based transistors, and [3] this remarkable performance deserves attention. If the band gap of a CNT can be tuned continuously, more robust CNT transistors will debut and it will diversify the applications of CNT transistors. However, tuning the band gap of semiconductors continuously [1], especially for carbon nanotubes, is an uphill challenge. The band gap of carbon nanotubes depends on discrete chirality numbers [1]. In order to get out of this dilemma, a continuously tunable length-dependent direct band gap of carbyne (in form of a linear carbon chain LCC) has been proposed for fine-tuning the energy barriers across semiconductor junctions [1,4]. Carbyne-based transistors have been demonstrated to have an outstanding current–voltage IV characteristic experimentally and the IV performance always depends on the applied strain or the size of carbyne [5], in which the band gap of carbyne can also be widely tuned by applying less than 10% strain [6]. The strain or length-triggered continuously tunable band gap of the carbyne inside a CNT may give a solution to widening the practical uses of CNT-based transistors. Unfortunately, the length of the carbyne during sample fabrication is unpredictable even though advanced manufacturing techniques are applied. There is no systematic routine that provides the theoretical input for optimizing the chain length of the carbyne, which hinders experimental bottom-up studies of the continuously tunable band gap.

The preparation of carbon chains with a length in the order of 20 atoms has been a difficult problem in the last decade [7,8]. To extend the chain length, Shi et al. encapsulated the carbyne with carbon nanotubes (CNTs) as a nanoreactor, resulting in carbon chains with up to 6000 atoms [9]. Based on the analysis of the carbyne@nanotube, it was argued that the van der Waals (VDW) force emanating from the carbon nanotube plays an important role in stabilizing the internal carbyne [4,9]. Inspired by the work of Shi et al., Chi Ho Wong et al. developed a Monte Carlo method (linear carbon chain (LCC) model) to study the stability of free-standing carbon chains laterally coupled by VDW forces at finite temperatures [10]. In the absence of carbon nanotubes, the direct action of the environment on the carbyne can be detrimental to the development of long chain lengths, but the kink structure [11,12] in a short carbyne can induce strong ferromagnetism [13]. One year later, Chi Ho Wong et al. succeeded in fabricating a ferromagnetic VDW-coupled free-standing carbon chain array with a length of ~100 atoms at room temperature assisted by the LCC model [13], where its ferromagnetism survives above 400 K. This observation is consistent with the argument of Shi et al. that the VDW interaction can be used to stabilize carbyne. On the other hand, several dopants have been proposed to enhance the magnetism of carbyne-based semiconductors with a local magnetic moment above 1.7 µB [13,14].

Since the discovery of the 400 K ferromagnetism in carbyne, carbyne may become a potential material to study the magnetic interface/edge problems for the development of room-temperature spintronic devices. To take spintronic nanodevices to the next level, edge magnetism [15] of semiconductors is the most promising route. The monoatomic structure makes carbyne an ideal candidate for studying the physics of edge magnetism, as it is much thinner than any unit cell thick nanowire or ultrathin zigzag nanoribbons of graphene [16,17]. From a scientific point of view, the spin–spin interaction of a monoatomic chain contributes to one of the types of boundary magnetism in a higher-dimensional Bravais lattice. If the chain length of a magnetic carbyne can be properly tuned [11,12,13], the length-dependent spin–spin interaction can be experimentally investigated down to the monoatomic scale, providing not only valuable scientific information for monitoring the interfaces of magnetic compounds for spintronics [18], but also insights into the interface problems of magnetic heterostructures [19] on an industrial scale.

In spite of technological and scientific advances, a tiny change in experimental parameters can fragmentize carbyne during sample fabrications. Theoretical guidelines are needed to control the carbyne length within a targeted range. However, the LCC model can only compare the stability of fixed-length carbyne [10], which is not designed for a variable-length condition. The kink structuring of a carbyne chain simulated by the LCC model remains continuous no matter how long the chain is. A similar problem can be noticed in DFT software. Chain breakage is not observed in an isolated carbyne even though the carbyne is very long, and the entire isolated chain remains linear, surprisingly, after applying geometric optimizations. Worse still, the DFT study of finite-length carbyne is limited to only ~100–200 atoms due to prohibitive computational cost [20]. For example, a ~6000-atom-long carbyne surrounded by a CNT (over ~100,000 atoms per unit cell) may not be a realistic task for DFT software [21]. To speed up the computational progress, using DFT software to calculate an infinitely long LCC with few atoms per unit cell is inevitably a popular option [14,22]. DFT methods are ideal for situations at 0 K [23]. DFT results are usually representative unless the materials are extremely unstable at finite temperatures. If a long linear carbon chain (LCC) dissociates at room temperature, a transfer function should be imposed on DFT methods to align theoretical results with experimental observations. Better software is needed to monitor carbyne in variable-length conditions. Otherwise, it is unclear how the sample’s quality affects the growth of internal LCC, and how the fragmentation of LCC takes place.

In view of the above problems, we developed a Monte Carlo algorithm to predict the stability of carbyne@nanotubes at finite temperatures with reasonable computational effort. A stochastic process is monitored to predict the stability of the carbyne@nanotubes up to 300 K. Parametric studies of the composite focus on the external parameters (e.g., pressure, temperature) and the internal parameters (chain length of carbyne, chirality, porosity of the carbon nanotube, etc.). Molecular modeling for the formation of fragmentized carbyne will debut in this work. After the Monte Carlo algorithm is introduced, we will propose a potential LCC@CNT composite to vary the band gap continuously in the CNT-based transistors.

## 2. Methods

### 2.1. Hamiltonian

The Hamiltonian of the carbyne@nanotube with length L is:

(1) $$\begin{array}{cc} H=e^{-\frac{T}{T_{\mathrm{bj}}}} & \left(\overset{N}{\underset{n=1,3,5}{\sum }}\left|E_{n,j}-E_{1}\right|e^{-\frac{l_{n}-l_{n,j}^{\mathrm{eq}}}{0.5l_{n,j}^{\mathrm{eq}}}}+\overset{N}{\underset{n=2,4,6}{\sum }}\left|E_{n,j^{\prime }}-E_{3}\right|e^{-\frac{l_{n}-l_{n,j^{\prime }}^{\mathrm{eq}}}{0.5l_{n,j^{\prime }}^{\mathrm{eq}}}}\right)+e^{-\frac{T}{T_{\mathrm{bj}}}}\overset{N}{\underset{n=1,2,3}{\sum }}J_{A}e^{-\frac{l_{n}-l_{n,j}^{\mathrm{eq}}}{0.5l_{n,j}^{\mathrm{eq}}}}{\left(\cos\theta +1\right)}^{2}\\ & \;\;-4\varphi \overset{2\pi }{\underset{\phi =0}{\sum }}\overset{N}{\underset{n=1}{\sum }}\left[{\left(\frac{\sigma }{r}\right)}^{6}-{\left(\frac{\sigma }{r}\right)}^{12}\right] \end{array}$$

where N is the total number of atoms in the carbon chain and T is the surrounding temperature. The C−C, C=C, and C≡C bond energies are E_1_ = 348 kJ/mol, E_2_ = 614 kJ/mol, and E_3_ = 839 kJ/mol, respectively [10]. The type of covalent bond is proposed by stochastic variables j and j’. For example, E_n,j_ (n = 100, j = 3) refers to the 100th carbon atom (n = 100) forming a triple bond (j = 3) with respect to the 99th carbon atom (n − 1 = 99). The temperature of bond dissociation is T_bj_ = E_j_/k_B_, where k_B_ is the Boltzmann constant [10]. The chain-stability factor is defined by $e^{-\frac{l_{n}-l_{n,j}^{\mathrm{eq}}}{0.5l_{n,j}^{\mathrm{eq}}}}$ [10].

The bond distance l is computed in Cartesian coordinates. The longitudinal axis refers to the x-axis, while the y- and z-axis form a lateral plane. The C−C, C=C, and C≡C bond lengths on the ground state are $l_{n,1}^{\mathrm{eq}}$= 154 pm, $l_{n,2}^{\mathrm{eq}}$= 134 pm, and $l_{n,3}^{\mathrm{eq}}$= 120 pm, respectively [10]. For the kink term, the bond angle (or pivot angle) between three adjacent carbon atoms is θ. A linear carbon chain means θ = 180 degrees, where the angular energy J_A_ is ~600 kJ/mol [10]. The van der Waals interaction between the carbon nanotube and the internal chain is $E_{\mathrm{vdw}}=-4\varphi \sum_{\phi =0}^{2\pi }\sum_{n=1}^{N}\left[{\left(\frac{\sigma }{r}\right)}^{6}-{\left(\frac{\sigma }{r}\right)}^{12}\right]$ [10]. The $\sum_{\phi =0}^{2\pi }$ sums up VDW interaction along the angular plane ϕ of a CNT and r is the radial separation between the LCC and the CNT. By considering $L\frac{{\mathrm{dE}}_{\mathrm{vdw}}^{2}}{{\mathrm{dL}}^{2}}=\frac{1}{\zeta }$ and $\frac{\partial E_{\mathrm{vdw}}}{\partial r}=0$ [10], the calculated VDW constants are σ~1.2 × 10^−10^m and φ~8 × 10^−23^J [10], where ζ is the isothermal compressibility. VDW interaction is the only coupling between the LCC and the CNT.

### 2.2. Initial Condition

**Figure 1:**
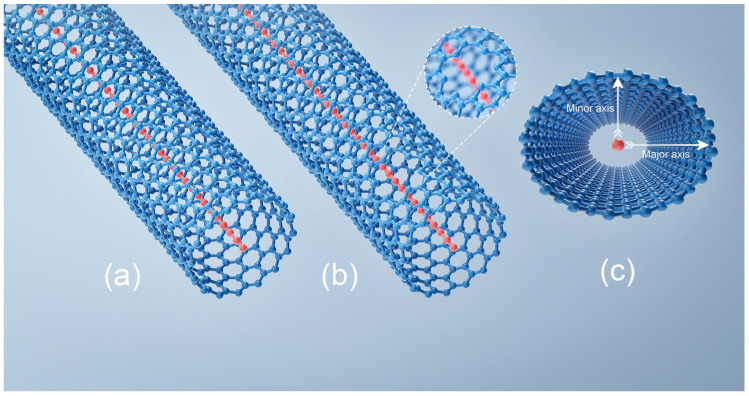
The carbon nanotube is marked by blue. The red internal chain refers to the carbyne. (**a**) Carbyne@nanotube. (**b**) Porous carbyne@nanotube. (**c**) The cross-section of a radially compressed carbyne@nanotube. The compression is along the minor axis.

A 6000-atom-long LCC (unless otherwise specified) surrounded by a single-walled carbon nanotube (SWCNT) is studied, which is abbreviated as LCC@(N_c_,M_c_)CNT, as shown in Figure 1a. The LCC@(N_c_,M_c_)CNTs are spaced by ~0.5 nm laterally in a parallel array, where (N_c_,M_c_) refers to the chirality of CNT. After applying geometric optimization to a periodic LCC@(N_c_,M_c_)CNT at the GGA level under the LAPW method (Muffin radius ~1.3 au; the total number of k-points in the Brillouin zone ~200) [24,25], the repeated unit of a CNT is imported in our Monte Carlo simulation. The initial bond length of the internal LCC (cumulene phase: consecutive double bond) is 134 pm. The lengths of the LCC and (N_c_,M_c_)CNT are equal. The length of the (N_c_,M_c_)CNT in our Monte Carlo simulation can be scaled using the repeated unit. The porous carbyne@nanotube and the radically compressed carbyne@nanotube are drawn schematically in Figure 1b,c, respectively.

### 2.3. Algorithm

During the Monte Carlo iterations, the atomic coordinate and the type of covalent bond in the carbon chain are amended at finite temperatures. In each Monte Carlo step (MCS), we randomly select an atom in the carbon chain and then calculate the initial Hamiltonian. 

To propose the trial types of covalent bonds at each MCS, we assign a random number $0\leq R_{\mathrm{bond}}\leq 1$ in Table 1. The proposed types of covalent bonds depend on the value of R_bond_. For example, the selected nth atom forming two double bonds with its nearest neighbors is expressed as [=C=]. If [=C=] is detected and R_bond_ = 0.8, the trial type of covalent bond is [−C−].

Based on the metropolis algorithm [26] and the LCC model [10], we propose a trial range for the spatial fluctuation at each MCS. Three random numbers R_x_, R_y_, and R_z_ from 0 to 1 are generated, respectively. If R_x_ > 0.5, the selected atom moves longitudinally in the +x direction. Otherwise, it moves in the -*x* direction. The same cut-off value of 0.5 is also applied to the sign convention along the *y*- and *z*- axis. After the trial directions are proposed, the trial fluctuations (*δx, δy, δz*) of the carbon atom are $\delta x=\pm v<\delta t>R_{c}$ and $\delta y=\delta z\sim \frac{k_{B}T}{\langle E_{1}+E_{2}+E_{3}\rangle }\delta x$, where the free particle velocity is $v=\sqrt{\frac{k_{B}T}{M}}$ [27]. The average scattering time $\langle \delta t\rangle$ and the mass of a carbon atom M are ~10^−13^ s and 19.9 × 10^−27^ kg, respectively [10]. The random number $0<R_{c}<1$ is to fine-tune the spatial fluctuation at each MCS.

Hooke’s factor, $f\left(Hooke\right)=\frac{K_{break}\left(leq_{max}()\right)}{K_{carbyne@CNT}\left({\left.l_{carbyne@CNT}\right|}_{0K}-\langle l_{eq}\rangle \right)}$, acts as a mean-free-path MFP controller to adjust the relative dynamics ($\delta x\cdot f\left(Hooke\right)$, $\delta y\cdot f\left(Hooke\right)$, and $\delta z\cdot f\left(Hooke\right)$) from a chain-breaking state to a crystalline form. Hooke’s factor mimics the situation where a stable carbyne@nanotube should have a shorter MFP and narrower range of spatial fluctuation than an unstable one. The numerator of $f\left(Hooke\right)$ marks the elastic force when an isolated LCC is elongated from a ground state to a breakpoint [27]. The breakpoint refers to the formation of the maximum C−C length $l_{max}$~1.73 Å where the MFP is greatest [28]. The ground state of an isolated LCC (cumulene phase) has an average bond length of $\langle l_{eq}\rangle$~1.34 Å. The spring constant of an LCC at the breakpoint is $K_{break}$. The denominator of $f\left(Hooke\right)$ monitors the elastic force of a very long LCC protected by a CNT relative to the same $\langle l_{eq}\rangle$ [27]. The spring constant and average bond length of the internal LCC at 0 K is $K_{carbyne@CNT}$ and ${\left.l_{carbyne@CNT}\right|}_{0K}$, respectively. In our DFT simulation, the GGA-PBE functional is chosen [24,25]. If we set the bond length to be 0.173 nm, we obtain the $K_{break}$ of an isolated infinitely long LCC chain. The $K_{carbyne@CNT}$ refers only to the spring constant of the internal LCC along the chain axis.

After the trial range of spatial fluctuations and trial types of covalent bonds are proposed, we estimate the trial Hamiltonian. If the trial Hamiltonian is less positive (or more negative) than the initial Hamiltonian, the trial states are accepted [26]. Otherwise, the system returns to the previous states [26]. Thermal excitation is another opportunity to accept or reject the trial states in parallel. If the random number $0\leq R_{B}\leq 1$ is smaller than the Boltzmann factor $e^{\frac{-\Delta H}{k_{B}T}}$, the trial states are accepted [26]. Otherwise, the system discards the trial states. The Monte Carlo process continues until the Hamiltonian vs. Monte Carlo steps approach a flat line (~250,000 steps). We average the data in the range of 230,000≤MCS≤250,000. We impose a boundary condition that the location of the 1st atom in the LCC is fixed and the covalent bond between the 1st and 2nd atoms in the LCC is always the C−C bond. The initial condition is executed again at each temperature. The octet rule [27] is applied.

### 2.4. Pre-Calibration

The average bond distance of a finite length LCC in the CNT ${\left.l_{carbyne@CNT}\right|}_{0K}$ is unknown before the simulation begins. To determine a reasonable trial value of ${\left.l_{carbyne@CNT}\right|}_{0K}$, we use the recently presented LCC model [10] to generate a long LCC of known N. We calculate the average bond distance of the LCC ${\left.l_{carbyne}\right|}_{501K}$ above the Peierls transition temperature at ~500 K [10,22], at which the polyyne phase forms automatically. Since $K_{carbyne@CNT}$ and $\langle l_{eq}\rangle$ refer to the situation at 0 K, the ${\left.l_{carbyne}\right|}_{0K}$ is estimated with the help of $\frac{{\left.l_{carbyne}\right|}_{501K}-{\left.l_{carbyne}\right|}_{0K}}{{\left.l_{carbyne}\right|}_{0K}}=\alpha \Delta T$, where the thermal expansion coefficient is α = 7 × 10^−5^ K^−1^ [10,29].

To obtain the value of ${\left.l_{carbyne@CNT}\right|}_{0K}$, we perform a pre-calibration by substituting the trial ${\left.l_{carbyne}\right|}_{0K}$ into Hooke’s factor. Then, we perform the Monte Carlo simulation (Section 2.2 and Section 2.3) of LCC@(N_c_,M_c_)CNT at T~0 K with the trial Hooke’s factor. During the Monte Carlo iterations of the internal LCC, the effect of a CNT tunes the average bond length from ${\left.l_{carbyne}\right|}_{0K}$ to ${\left.l_{carbyne@CNT}\right|}_{0K}$. After the pre-calibration, we substitute ${\left.l_{carbyne@CNT}\right|}_{0K}$ to Hooke’s factor. With the calibrated Hooke’s factor, our simulation tool is ready to study the stability of LCC@(N_c_,M_c_)CNT.

## 3. Results

**Figure 2:**
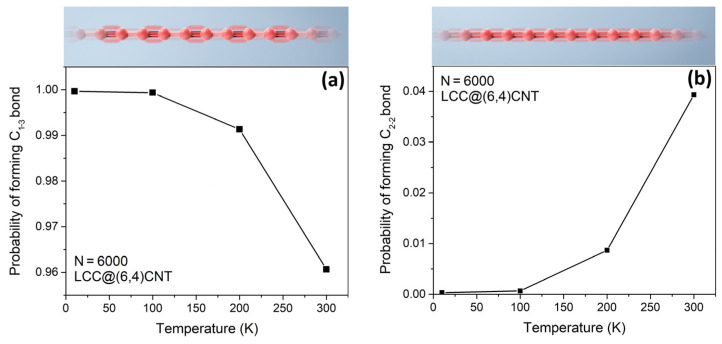
An LCC encapsulated by a (6,4)CNT upon heating. The LCC is made up of 6000 atoms. (**a**) The probability of maintaining a polyyne phase in the carbon chain. The C_1–3_ bond is defined as the formation of a single bond and a triple bond alternatingly. The red chain shows the structure of polyyne schematically. (**b**) The probability of maintaining a cumulene phase in the carbon chain. The C_2–2_ bond is defined as the formation of consecutive double bonds. The red chain refers to the schematic structure of cumulene.

Thermal excitation from 0 K to 300 K has changed the proportion of polyyne and cumulene phases in Figure 2. We found that the probability of forming a C_1–3_ bond (polyyne phase) in the internal LCC decreases from 1.00 to 0.96 upon heating. Due to the growth of the polyyne phase, the probability of forming a C_2–2_ bond (cumulene) correspondingly increases to ~0.04. The formation of C_1–2_ ([=C−] or [−C=]) and C_1–1_ ([−C−]) bonds is rare. The most energetically favorable phase of the internal LCC is still polyyne, even when the temperature is 300 K [9]. After the pre-calibration process at T~0 K, the average bond length of the internal LCC is ~1.3 Å and the bond length alternation is above 99.9%.

**Figure 3:**
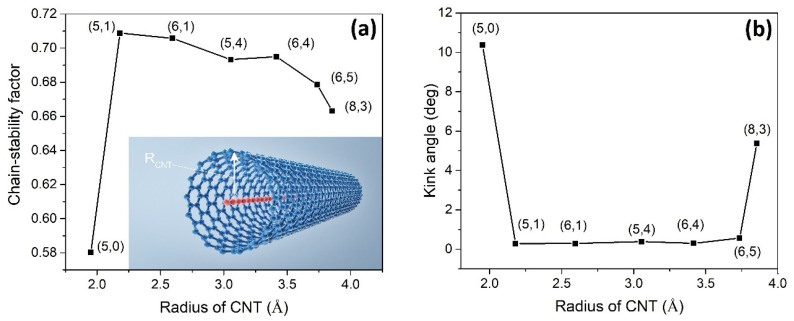
(**a**) The stability of the 6000-atom-long carbyne@nanotube depends on the radius of carbon nanotube R_CNT_ and the chirality (N_c_,M_c_). The red chain is LCC while the blue tube is CNT. The temperature of the thermal bath is 300 K. For the chain stability factors near 0.7, the average bond lengths of the LCCs range from ~1.3 to ~1.4 Å. (**b**) The average kink angles of the internal LCCs are plotted accordingly. A kink angle of zero (or pivot angle = 180°) means that the chain is linear. The standard deviation of the LCC@(6,4)CNT is as low as ~0.1.

As illustrated in Figure 3a, the stability of the carbyne@nanotube depends on the radius of the CNT associated with its chirality (N_c_,M_c_) [4,9]. In the carbyne@nanotube experiment [9], (6,4) and (6,5) CNTs were the most abundant components. At a constant temperature of 300 K, our simulation data show that the (6,4)CNT should have a longer LCC than (6,5)CNT. Although the experiment [9] does not contain (5,0), (5,1), (6,1), and (5,4)CNTs, we used these samples in our simulation. The LCC@(5,1)CNT has the highest chain stability factor among the others, whereas the chain stability of LCC@(5,0)CNT is the worst. Figure 3b demonstrates how the kink angles of the internal LCC are affected by the geometry of the CNTs. The average kink angle of LCC@(5,0)CNT is much higher than the others. It can be observed that the higher the chain stability factor, the lower the average kink angle of the LCC.

**Figure 4:**
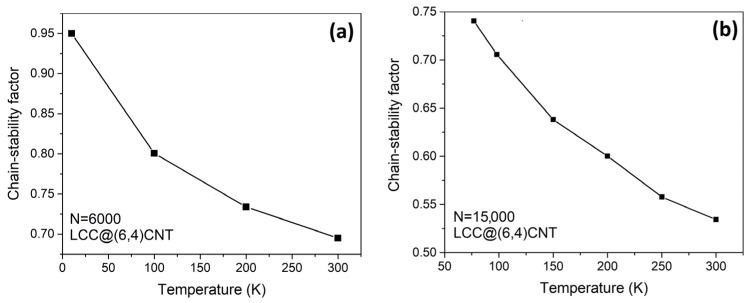
An LCC encapsulated by a (6,4)CNT during heating. The atomic fluctuations of the LCC depend on the chain length and temperature. (**a**) The LCC consists of 6000 atoms. (**b**) The LCC contains 15,000 atoms.

**Figure 5:**
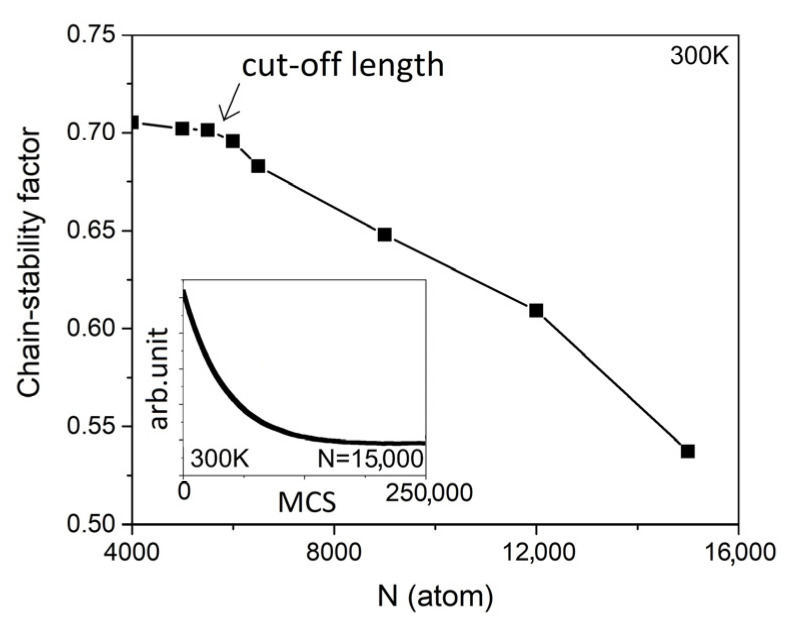
The chain stability factor is a measure of atomic fluctuations of LCC@(6,4)CNT. The reduction in the chain stability factor is more pronounced at N > 6000. The inset shows that our sample reached an equilibrium state at the Monte Carlo steps exceeding ~180,000.

The chain stability of the internal LCC consisting of 6000 atoms and 15,000 atoms is reduced by thermal excitation, as demonstrated in Figure 4. The LCC with 6000 atoms in length has a higher chain stability factor $e^{-\frac{l_{n}-l_{n,j}^{eq}}{0.5l_{n,j}^{eq}}}$ than the LCC with 15,000 atoms in length, regardless of the temperature. Comparing the chain stabilities in both cases, it can be interpreted that the atomic fluctuations of the 6000-atom-long LCC are always lower at the same temperature. The chain stability of the carbyne@nanotube in different lengths is compared in Figure 5. At T = 300 K, the chain stability factor gradually decreases when N is less than 5500. However, at N > 6000, a rapid reduction in the chain stability factor is observed when we halve the cut-off length of the LCC to N = 5750. The inset in Figure 5 refers to the average energy of the internal LCC as a function of the Monte Carlo iterations. 

**Figure 6:**
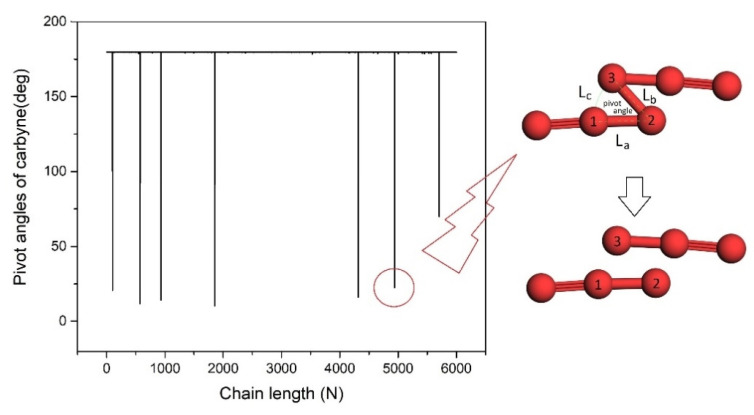
The pivot angle distribution of the carbon chain enclosed by a ~4% radially compressed CNT. Seven critical kink structures with shape pivot angles are observed. One of the critical kink structures is drawn schematically (not to scale). L_c_, L_b_, and L_a_ form a pivot angle. L_b_~1.73 Å and 1.5 Å < L_a_ < 1.6 Å are always observed in all the critical kink structures. Formation of 7 + 1 = 8 carbyne fragments is expected at an instantaneous moment of chain breakage in test 1 (Table 2). The electrostatic repulsion between the sites ‘1’ and ‘3’ is stronger than triple-bond strength.

A compressive strain in the radial direction to the LCC@(6,4)CNT deteriorates the chain stability. The chain stability factor drops from 0.69 to 0.68 at a compressive strain of 2%. However, when the compressive strain reaches 4% in test 1 (Table 2), the formation of seven extremely sharp pivot angles within ~10^o^ < pivot angle < ~60° at n = 103rd, 576th, 932nd, 1853rd, 4318th, 4943rd, and 5705th atom are observed in Figure 6. The critical kink structure around n = 4943rd (marked by a red circle) is drawn. The carbon–carbon distances nearby the sharp kink points always reach ~1.73 Å. The carbon atoms at the sharp kink points are mostly connected by C−C−C. More samplings are shown in Table 2 where we repeat the studies of 4% radial compression in test 2 and test 3.

The porosity of the (6,4)CNT harms the chain stability of the internal LCC, as demonstrated in Table 3. The introduction of a tiny amount of vacancy defects is sufficient to significantly shorten the cut-off length to N~5600. In Table 4, the cut-off length of the LCC@(N_c_,M_c_)CNT in various chiralities at 300 K is investigated. The internal LCC in the (5,1)CNT is the longest. The internal LCC in the (6,4)CNT is ~10% longer than that in a (6,5)CNT. Based on the data in Table 5, it is possible to extend the internal LCC to a length of 15,000 atoms. However, the sample should be cooled to T~120 K. Our model expects that the internal LCC in the (6,4)CNT should increase by less than 10% at 273 K.

## 4. Discussion

Although the cumulene phase should dominate in an isolated LCC [22], the polyyne phase is energetically more favorable in the LCC@(N_c_,M_c_)CNT [9]. According to Figure 2, the polyyne phase fades upon heating because the atomic fluctuations are more severe at high temperatures. This process can be controlled by the Boltzmann factor [26], which more readily accepts the trial type of covalent bonds at high temperatures [10]. The formation of [−C=], [=C−], and [−C−] is rare, confirming that our Monte Carlo simulation effectively monitors the process of energy minimization. For instance, the energy difference between [−C≡] and [=C=] is |348 + 839 − (614 + 614)| = 41 kJ/mol per site. However, the energy difference (per site) between [−C≡] and [−C=], and the energy difference (per site) between [−C≡] and [−C−] are 225 kJ/mol and 491 kJ/mol per site, respectively. Hence, when the sample heats up, the LCC prefers to accept the trial state [=C=] rather than [−C=], [=C−], and [−C−].

We study the stability of the internal LCC as a function of R_CNT_ in Figure 3a. While the chain length is fixed, the optimization process focuses only on the radial direction of the CNT. An isolated long LCC is always unstable in a room temperature environment due to strong atomic fluctuations [5,6,7,30,31]. The kink angle is a measure of the atomic fluctuations in the LCC. The kink angle of the LCC in Figure 3b can be used to judge whether the CNT provides appropriate VDW protection or not. The average kink angle of the internal LCC in (5,1)CNT is only 0.3 degrees (or pivot angle = 179.7 degrees) in Figure 3b. This explains why the LCC surrounded by (5,1)CNT has the highest chain stability factor where self-dissociation or chain breakage is less likely. Our simulation result is consistent with the experimental results that the internal LCC in a (6,4)CNT is longer than that in a (6,5)CNT [9].

The chain stability factor of the LCC@(6,4)CNT increases upon cooling (see Figure 4). At low temperatures, the movement of carbon atoms is inactive (i.e., the amplitude of atomic vibration is small). The carbon atoms in the LCC tend to remain close to their equilibrium positions due to reduced atomic fluctuations at lower temperatures. (i.e., $l_{n}$~$l_{n,j}^{\mathrm{eq}}$) that increases the chain stability factor $e^{-\frac{l_{n}-l_{n,j}^{\mathrm{eq}}}{0.5l_{n,j}^{\mathrm{eq}}}}$. At high temperatures, the strength of the covalent bond between carbon atoms may not be sufficient enough to resist atomic fluctuations, leading to chain breakage or self-dissociation of the LCC. In other words, the minimization of atomic fluctuations in the LCC is a way to maintain the effectiveness of covalent bonds in order to avoid chain breakage or self-dissociation. Therefore, it is feasible to extend the length of the LCC beyond N~6000, but precautions must be taken to minimize atomic fluctuations, such as implementing a cooling mechanism. A cooler environment can effectively limit the atomic fluctuations, reinforcing the covalent bond strength and reducing the risk of chain breakage or self-dissociation. As atomic fluctuations can be influenced by temperature continuously, and since the maximum length of the LCC is dependent on these atomic fluctuations, it may build a relationship between the maximum LCC length and temperature.

If all covalent bonds in the LCC are stable, the LCC with a length of 15,000 atoms should have weaker atomic fluctuations than the LCC with a length of 6000 atoms. However, extensive studies of an LCC show that the elongation of an LCC always leads to chain termination or self-dissociation [4,5,6,7,9,30,31,32,33]. So far, the formation of all stable covalent bonds in an extremely long LCC has not been observed experimentally (48 carbon atoms at most). To make a longer LCC unstable in our model, Hooke’s factor is used to tune the trial range of atomic fluctuations. When our previous LCC model is run this time, the maximum bond length of an isolated LCC calculated by the thermal expansion coefficient being set to $l_{c-c}+l_{c-c}\alpha \Delta T$ ~1.56 Å manually. In this case, all covalent bonds are stable and eventually ${\left.l_{carbyne}\right|}_{0K}$ is shorter in a longer LCC.

The accuracy of the thermal expansion coefficient in our model was justified by Costa et al. [29]. After the substitution of ${\left.l_{carbyne}\right|}_{0K}$ into the pre-calibration process, the trial Hooke’s factor increases for a longer LCC. By setting the maximum bond length in this carbyne@nanotube model to ~1.73 Å [28], we impose a boundary condition that chain breaking is not allowed even if two carbon atoms are ~1.73 Å apart. Then, our Monte Carlo system can force the atoms to form unstable covalent bonds at any chain length L in excited states. The unstable covalent bonds associated with large atomic fluctuations outweigh the effect of stabilization toward the bulk state. Therefore, the LCC with a length of 15,000 atoms (Figure 4b) has a lower chain stability factor than the LCC with a length of 6000 atoms (Figure 4a). Without this boundary condition, we cannot adequately compare the stability of the different LCC@(Nc,Mc)CNT types at the same length.

Although Hooke’s factor is designed to be semi-empirical, our simulation can mimic the stability of a long LCC that is consistent with experimental observations, with the cut-off length (5750-atom-long) in Figure 5 being comparable to the experimental data (~6000-atom-long) [9]. Indeed, Hooke’s factor is just a ratio of velocity, because any elastic force multiplied by $\langle \delta t\rangle /M$ gives a unit of velocity [27]. Therefore, $v\cdot f\left(Hooke\right)$ can be read as an adjustment of the trial velocity. If a carbon chain is going to break [28], Hooke’s factor in a local region approaches 1, so using the free-particle velocity as the maximum velocity at the trial state would be appropriate [27]. The random number $R_{c}$ refines the spatial fluctuations to aid the process of energy minimization.

In our simulation, a single-walled CNT is used instead of a double-walled CNT to protect the LCC. A double-walled CNT mainly exerts influence on the internal LCC via the inner wall and the influence of the outer wall is very small, which was observed in the measurement of radial breathing modes [9]. Lei Shi et al. conducted the analysis of radial breathing modes (RBM) of the double-walled CNT experimentally, where only the RBM peaks corresponding to the inner CNT are split and enhanced significantly [9]. This indicates that the inner CNT is a dominant source interacting with the internal LCC [9]. Therefore, our simulations may not need to take the outer wall into account which mainly prevents the inner wall from collapsing (or maintaining a good shape) under perturbations only [4,9]. Our DFT simulation runs a parallel array of LCC@(N_c_,M_c_)CNT with a lateral spacing of 0.5 nm, and the lateral wall-to-wall interaction is enough to stabilize the single-walled CNT in a good shape. The cut-off length of the LCC@(6,4)CNT is slightly underestimated in our model. There are some sources of error. The ${\left.l_{carbyne@CNT}\right|}_{0K}$ in Hooke’s factor is obtained from the pre-calibration process for N > 4000, but the $K_{carbyne@CNT}$ is the spring constant of an infinitely long LCC. Since the computational cost of a DFT simulation may not be realistic when the size exceeds 4000 atoms per unit cell [21], our model assumes that the mechanical properties of the LCC consisting of more than 4000 atoms are likely to move toward the bulk state [34,35]. Moreover, the iterative process of our Monte Carlo simulation is not applicable to the atoms in CNTs, as experimental results show that the atomic vibrations of CNTs are much weaker than those of the internal LCCs. Based on these experimental findings, we can assume that the atoms in the CNTs are relatively at rest. Although the cut-off length is slightly underestimated by these approximations, the computational cost becomes affordable. The inset in Figure 5 confirms that 250,000 Monte Carlo steps are sufficient to drive the system to equilibrium, even if the longest sample is chosen.

The shape of the CNT plays an important role in stabilizing the carbyne@nanotube. The compressive strain changes the CNT from a circular to an elliptical shape. Although the elliptical CNT has a slightly more negative VDW interaction along the minor axis (shorter axis), the VDW interaction along the major axis (longer axis) is much weaker. This is the reason why radial compression destabilizes the LCC@(6,4)CNT. On the other hand, a stronger spatial fluctuation in the internal LCC is observed in porous CNTs. If the selected atom in the internal LCC sums up the negative VDW terms along the angular plane ϕ, the missing carbon atoms in the porous CNT cannot turn the VDW force more negatively, and thus maintaining a good sample quality of a CNT is important to prolong the internal LCC.

Our Monte Carlo results are consistent with several experimental observations. (1) The LCC in a (6,4)CNT is more stable than the LCC in a (6,5)CNT [9]. (2) The quality of the CNT affects the stability of the internal LCC [4,9]. (3) The cut-off length of the LCC@(6,4)CNT is about ~6000 atoms long [9]. (4) Polyyne is the dominant phase in LCC@(N_c_,M_c_)CNT at room temperature [9]. These Monte Carlo results encourage us to predict the cut-off length of the internal LCC surrounded by different CNT types in Table 4. Based on our Monte Carlo model, the LCC surrounded by a (5,1)CNT should be the longest among the others because the atomic fluctuation is the weakest. The effect of free radical electrons [27] in the LCC is ignored in our model since the probability of forming a polyyne phase is close to 1. When the trial kink angle is large, the selected carbon atom experiences a non-uniform VDW interaction along the angular ϕ plane. The non-uniform VDW interaction makes the Hamiltonian more positive, which is consistent with the data in the compressed CNT. For example, the selected carbon atom in the LCC orthogonally shifted by 0.01 nm within the (5,4)CNT, increasing the VDW energy by 2% more positive.

Our model assists in the exploration of the chain-breaking process and the growth mechanism of carbyne. An overlength carbyne (exceeding the cut-off length) always breaks into fragments during sample fabrication due to chemical instability [33]. What happens to an overlength carbyne before it breaks naturally? To answer this question, an overlength carbyne needs to be generated in our simulation model to “visualize” the instantaneous atomic fluctuations right before it breaks. A carbon chain made up of 5500–6000 atoms (~780 nm) ought to be stable inside a (6,4)CNT. When the LCC@(6,4)CNT is compressed by ~4% (Figure 6), the ~780 nm long internal carbyne becomes overlengthened which may be fragmentized. Nair et al. [34] justified that a mechanical strain of at least 5% is needed to break a finite-length carbyne. In other words, the carbon chain should break when the C−C bond length reaches 1.5 Å× (1 + 5%) ~1.6 Å. After the 6000-atom-long LCC@(6,4)CNT is compressed, the seven sharp kink structures are always connected by the C−C bond lengths above 1.6 Å, which may initiate chain breakage. Hence, an effective covalent bond between sites ‘2’ and ‘3’ (Figure 6) should vanish. The reestablishment of an effective covalent bond between sites ‘1’ and ‘3’ is unlikely to occur because our ab-initio calculation shows that the carbon chain with a pivot angle around 20 degrees generates a local electrostatic repulsion at least two times stronger than the triple-bond energy. In addition, for the case of a pivot angle ~50 deg (Figure 6), the carbon atom in site ‘3’ should be unable to form an effective covalent bond with the CNT wall because the radial separation between the carbon atom in ‘site 3’ and the CNT wall is still as large as 1.96 Å. As a result, the LCC in the compressed (6,4)CNT is probably fragmentized into eight pieces during test 1, where the average size of the carbyne fragment is 780 nm/(7 + 1) = 97 nm. The eight short carbyne chains should consist of 103 − 1=102 atoms, 576 − 103 = 473 atoms, 932 − 576 = 356 atoms, 1853 − 932=921 atoms, 4318 − 1853 = 2465 atoms, 4943 − 4318 = 625 atoms, 7505 − 4943 = 762 atoms, and 6000 − 5705 = 295 atoms, respectively. Our model can be used to forecast where the long carbon chain should break at finite temperatures [33] and the instantaneous atomic configuration of the carbyne right before it breaks. Our model does not probe the further atomic movement of carbyne fragments after the long chain breaks. Based on Ref. [34], the mechanical properties of carbyne show a size effect up to N~20. If the carbyne fragment is less than 20 atoms long, the atomic spring constant in Hooke’s factor should be revised. However, the shortest carbyne fragment formed by compression still contains over 100 atoms and, hence, Hooke’s factor is still applicable to monitor the fragmentation of the carbyne.

As an example, we investigate if scientists want to use LCC@(5,5)CNT in the transistor for an emerging continuously tunable band gap, where the radius of (5,5)CNT of 0.339 nm is comparable to the radius of (6,4)CNT of 0.341 nm. A metallic (5,5)CNT exists [36] and the precise control of chiral angles in a CNT enters a new era [37]. When LCC@(5,5)CNT is unstrained, the cut-off length of the LCC is ~800 nm. After LCC@(5,5)CNT is strained by 1% longitudinally, the cut-off length drops to ~750 nm. To ensure the internal carbyne is stable during the operation, the maximum length of the unstrained LCC@(5,5)CNT should not exceed ~750 nm. The ~750 nm long carbyne in the (5,5)CNT is stable at 1% strain. When the LCC@(5,5)CNT is unstrained, the stability of the LCC is even stronger, based on the analysis in Figure 5. Hence, neither the 1% strain nor the unstrained condition destroys the internal carbyne, and more encouragingly, the 1% strain should change the band gap of the carbyne by ~15% continuously [6], which provides precise control in the energy barriers in the transistors.

The low computational cost of our Monte Carlo model is credited to two approximations. The first approximation is that the atomic positions of the nanoreactor (finite-length CNT) in our Monte Carlo model are obtained by scaling the repeating unit in the ab-initio calculations. This speeds up the computational progress because a time-consuming geometric optimization process of a big supercell (over 100,000 atoms in a non-repeating unit) is avoidable. To validate this approximation, we consider the length dependence of the Raman spectrum of a finite-length CNT where the size effect starts to pale for a CNT length longer than ~200 nm [38]. As the shape of the Raman spectrum is closely related to the atomic positions of materials and the minimum length of the nanoreactor CNT in our model is far above 200 nm, it is arguable that the first approximation will not create a large error in Monte Carlo results. The second approximation is that the Monte Carlo iterations are conducted to the internal chain only. This approximation can be validated by the experimental fact that the atomic fluctuations of the CNT are relatively at rest when compared to the internal chain [9]. With the second approximation, the size of a one-dimensional array in our Monte Carlo simulation is 4000–15,000 only, which reduces the computational time massively without creating a large error in Monte Carlo results. However, the appropriateness of using our Monte Carlo simulation for modeling carbyne is dependent on the particular scenario. For example, our Monte Carlo simulation may need modifications if it is asked to accurately predict the length of the LCC inside a very short CNT. In contrast, DFT software does not suffer from this issue because any super-cell function in DFT software is designed for studying nanomaterials approaching the size of quantum dots.

## 5. Conclusions

Our Monte Carlo model can be used to predict the cut-off length of the LCC encapsulated by a CNT at any temperature. Synthesis of the internal carbon chain with more than 6000 atoms is possible if the temperature and the type of the CNT are chosen correctly. The radius, chirality, and quality of the CNT have a great influence on the stabilization of the internal LCC. Radial compression on the carbyne@nanotube destabilizes the internal LCC. Apart from these, our investigation of the compressed carbyne@nanotube leads us to discover the mechanisms behind the chain-breaking process and the probable formation of carbyne fragments. Our model opens a new way to study the stability of carbyne in the size of 4000–15,000 atoms at finite temperatures, which is a steppingstone for experimental bottom-up studies of the edge magnetism from the monoatomic structure to a higher-dimensional Bravais lattice and continuously tunable energy barriers in the CNT-based transistors.

## Figures and Tables

**Table 1:** The trial state of a covalent bond.

(A) If [=C=] Is Detected	**Trial State**
$0\leq R_{bond}<0.33$	[≡C−] or [−C≡] in equal probability
$0.33\leq R_{bond}<0.66$	[=C−] or [−C=] in equal probability
$0.66\leq R_{bond}\leq 1$	[−C−]
(B) If [≡C−] or [−C≡] is detected	Trial state
$0\leq R_{bond}<0.33$	[=C=]
$0.33\leq R_{bond}<0.66$	[=C−] or [−C=] in equal probability
$0.66\leq R_{bond}\leq 1$	[−C−]
(C) If [−C−] is detected	Trial state
$0\leq R_{bond}<0.33$	[≡C−] or [−C≡] in equal probability
$0.33\leq R_{bond}<0.66$	[=C−] or [−C=] in equal probability
$0.66\leq R_{bond}\leq 1$	[=C=]
(D) If [=C−] or [−C=] is detected	Trial state
$0\leq R_{bond}<0.33$	[≡C−] or [−C≡] in equal probability
$0.33\leq R_{bond}<0.66$	[−C−]
$0.66\leq R_{bond}\leq 1$	[=C]

**Table 2:** The probable sizes of carbyne fragments in the ~4% compressed (6,4)CNT at 300 K.

Samplings	The Sizes of the Carbyne Fragments (Atoms)
Test 1	102, 473, 365, 921, 2465, 625, 762, 287
Test 2	546, 1650, 2251, 947, 138, 468
Test 3	71, 755, 164, 2003, 1081, 949, 207, 501, 269

**Table 3:** The effect of vacancy defects on the stability of the LCC@(6,4)CNT at 300 K.

Vacancy Defect (%)	Cut-Off Length of LCC@(6,4)CNT
0	5750-atom-long
2.5	5600-atom-long

**Table 4:** A prediction of the LCC lengths in different carbyne@nanotubes at 300 K.

Chirality (Nc,Mc)	Cut-Off Length of LCC@(Nc,Mc)CNT
(8,3)	4850-atom-long
(6,5)	5250-atom-long
(6,4)	5750-atom-long
(5,1)	7200-atom-long

**Table 5:** The predicted cut-off lengths of the LCC in a (6,4)CNT at different temperatures.

Cut-Off Length of LCC	Operating Temperature (K)
5750-atom-long	300
6600-atom-long	273
15,000-atom-long	122

## Data Availability

Data availability is possible upon reasonable request.
